# Systematic review of teacher well-being research during the COVID-19 pandemic

**DOI:** 10.3389/fpsyg.2024.1427979

**Published:** 2024-09-06

**Authors:** Millicent Aziku, Baohui Zhang

**Affiliations:** Faculty of Education, Shaanxi Normal University, Xi'an, China

**Keywords:** teacher well-being, systematic review, COVID-19 pandemic, research methodology, yearly distribution

## Abstract

**Introduction:**

The COVID-19 period posed great challenges to global education systems, especially teachers even after lock-down. Teachers' wellbeing has been a concern because they have to balance health with work. Since the role of teachers is pivotal in education, there is increased in research on their wellbeing status.

**Method:**

The current systematic review aims to analyze the distribution of research on teacher wellbeing from 2020 to mid-February 2024 using a quantitative method. It focuses on yearly distribution of studies, the research methods adopted by scholars, and the group of teachers investigated over the period. The PRISMA guidelines were followed, and 103 empirical studies were selected for the analysis.

**Results:**

The review shows notable increase in research, particularly in 2022 and 2023, representing 37.9% and 35.9% of studies, respectively. This suggests a growing interest in teacher wellbeing among educational researchers. The findings also indicate that researchers mostly adopted quantitative methods in form of surveys (79.6%) for studies on teacher wellbeing. However, there has been an increase in qualitative and mixed-methods research recently, with qualitative research accounting for 9.7% and mixed-method research accounting for 10.7%. The review also identified a greater focus on teachers in general than on specific group of teachers.

## 1 Introduction

Teacher well-being (TWB) is critical for building a sound teaching workforce because teaching is a high-stress profession (Yeh and Barrington, 2023). Research suggests that there is a need to promote the well-being of teachers because when teachers are mentally and physically fit, it can influence their job satisfaction and retention (Richter et al., 2022). Teacher well-being was defined by the World Health Organization (WHO) in 1946 as the state of complete physical, mental, and social well-being of teachers. Therefore, to ensure that teacher well-being is improved, the WHO emphasizes promoting the school climate, as the climate in which teachers work can affect their well-being (WHO, 2021). With an increased demand for quality teaching and learning at all levels of education (UNESCO, 2023), teacher well-being has become one of the major concerns for stakeholders in education (Granziera et al., 2023). Existing research shows that teachers' total well-being is essential to achieving quality education, as stress, burnout, and mental instability can lead to negative attitudes toward teaching (Zhu et al., 2021; Flores et al., 2022; Jeon et al., 2022). Other studies have associated teacher well-being with mentoring, in that school-based mentoring helps provide guidance and counseling support to teachers when faced with well-being issues (Burger et al., 2021). The responsibility of promoting teacher well-being does not rest only on schools and school leaders but also on society as a whole because teachers play a pivotal role in the sustainable development of societies (Wang and Hall, 2021; Dai et al., 2022; Yeh and Barrington, 2023).

### 1.1 On teacher well-being research

In recent years, interest in TWB has expanded in the field of educational and health research, especially because of the COVID-19 pandemic (McDonough and Lemon, 2022; Eblie Trudel and Sokal, 2023). Consequently, scholars have identified many individual-level, organizational-level, and relation-level factors that influence teacher well-being (MacIntyre et al., 2022; Chen et al., 2023). This is a good indication that scholars have started giving higher recognition to the role teacher well-being plays in improving quality education. Consequently, policymakers leverage the findings from extant research to respond to teachers' needs by organizing health support and professional development programs that target balancing work with personal life (Lau et al., 2022).

Researchers investigated teachers in different fields and levels over the years to gain a deeper understanding of TWB and expand research on the topic. This extensive research led to several reviews conducted on the topic, expanding the criticality of research on TWB. However, there is limited knowledge regarding the number of empirical studies conducted from 2020 to mid-February 2024. In light of this literature gap, the present systematic review explores the yearly research distribution on TWB from 2020 to mid-February 2024 and provides suggestions for educational researchers and policymakers.

#### 1.1.1 Previous systematic reviews on teacher well-being

The interest of researchers in teacher well-being has resulted in several systematic reviews by many other researchers, but with different focuses. For instance, Zhang et al. (2024) conducted a review on the topic of teacher well-being, focusing on articles published from 1968 to 2021, and recorded an expansion of studies, especially on the nature and effects of TWB. Several other systematic reviews were based on years prior to 2020, which indicates that little is known about research distribution on the topic, especially from 2020 to 2024. Meanwhile, a systematic review by Dreer and Gouase (2022) covered articles published in the early part of 2020 and was focused on interventions for TWB.

Relatedly, some other systematic reviews have analyzed TWB research in specific domains. For example, reviews implied that there were a considerable number of publications on early childhood teachers, as they are mostly faced with high levels of stress (Wilson et al., 2023). This finding indicates that a systematic review on TWB is still needed, focusing especially on the period 2020–2024, across all fields and among all teachers, to determine the yearly distribution of research in the field. According to Gray et al. (2017), there is a need to address teacher well-being issues to increase the interest of teachers in the profession.

Furthermore, the research methodologies mostly adopted by scholars in their studies on teacher well-being were examined in existing systematic reviews. For instance, according to Zhang et al. (2024), the quantitative research methodology was most commonly used to study TWB from 1968 to 2021, while there was an increase in the usage of both qualitative and mixed-method research from 2021. Moreover, a study reports that qualitative and mixed methods involving teachers at all levels were scarce (Katsarou et al., 2023). Several studies have suggested that teaching can be fulfilling, but it also brings about anxiety and places teachers under pressure as they struggle with various negative emotions (Fan, 2021). In light of these findings, various methods have been adopted to investigate teacher well-being.

### 1.2 Objectives

Despite many reviews on TWB, there are few reviews of the publications between 2020 and 2024, especially with a focus on the yearly distribution of research during the COVID-19 era and the group of teachers investigated over the years. Based on the findings from prior literature, this current systematic review through the quantitative method aims to analyze the distribution of teacher well-being research from 2020 to mid-February 2024, focusing on the yearly distribution of studies, research methodologies, and the participants investigated over the period using the following research questions:

Specific research questions

1. What is the yearly distribution of research on teacher well-being from 2020 to mid-February 2024?
2. What research methodologies were mostly adopted by researchers to investigate teacher well-being from 2020 to mid-February 2024?
3. Which group of teachers was most investigated by teacher well-being researchers from 2020 to mid-February 2024?

## 2 Method

**Figure 1:**
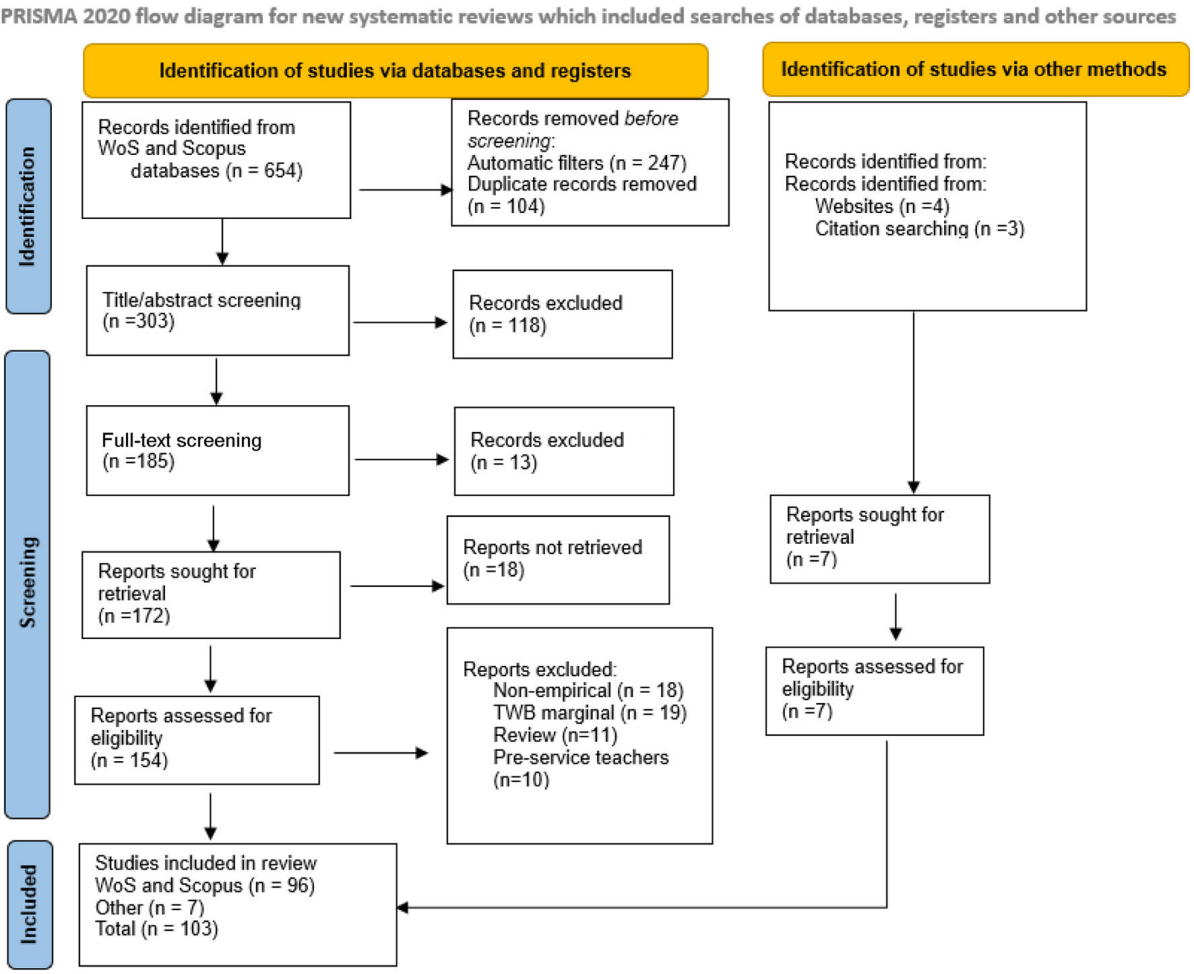
PRISMA flow diagram.

In the quest to answer the three research questions, the systematic review approach (identification, screening, and inclusion) was adopted for this current review to ensure that detailed steps are followed for a quality and standard systematic review, as suggested by the Preferred Reporting Items for Systematic Reviews (PRISMA) guidelines (Moher, 2009; Haddaway et al., 2022). The search for articles was primarily conducted on the Web of Science (WoS) and Scopus databases, and a snowballing strategy was used to locate studies from other databases (Google Scholar). The snowballing strategy involves searching for studies that were cited in other studies that the researchers assumed were relevant for the review. Each phase of the review process is shown in the PRISMA flow diagram, as shown in Figure 1, starting with the number of studies that were identified and included in the final stage.

### 2.1 Research strategy

Prior to conducting the systematic review, several inclusion criteria were formulated to ensure that the articles that would be retrieved aligned with the research objectives. First, journal articles were expected to be published from 2020 to 15th February 2024. This is necessary because, while there were several studies on teacher well-being prior to the pandemic (Salvagioni et al., 2017; Jeon et al., 2018; Skaalvik and Skaalvik, 2018), there is a need to understand whether the attention of scholars to the topic has increased during the onset of the pandemic and after schools reopened. In addition, several systematic reviews were already conducted in the years prior to 2020 (Hall-Kenyon et al., 2014; Slemp et al., 2020; Katsarou et al., 2023; Zhang et al., 2024), with few reviews on TWB between 2020 and 2024. Second, the research topic had to be relevant to teacher well-being or have dimensions or elements of teacher well-being. Third, the research participants needed to be in-service teachers only. Fourth, to ensure that the articles accessed are of high quality, they had to be peer-reviewed. This is essential because peer-reviewed papers undergo rigorous evaluation processes to ensure the validity, reliability, and scientific integrity of the methodology, analyses, and findings. Fifth, the research articles were required to be empirical and written in English.

In addition, the following exclusion criteria were established: First, if teacher well-being is not stated as the purpose of the study or at least as one of the purposes of the study. Second, studies that focus on participants other than in-service teachers. Third, studies that are not based on empirical research and are not in English. Fourth, if teacher well-being occupies a marginal segment of the research. In addition, to restrict the search to teacher well-being and prevent the inclusion of unrelated studies, the researchers used the following keywords as the search terms: “teacher well-being,” “educator well-being,” “instructor well-being,” “teaching” and “well-being,” and “teachers' well-being.” Using the quotation mark (“”) helps the researchers find the specific phrases in the literature.

### 2.2 Study selection

The present systematic review adopted the Preferred Reporting Items for Systematic and Meta-Analysis (PRISMA) guidelines for assurance of quality systematic review (Moher, 2009; Haddaway et al., 2022). Three steps were adopted to ensure an appropriate procedure for the inclusion of retrieved articles in the review: identification, screening, and inclusion (Haddaway et al., 2022). The first researcher identified a total of 523 and 131 studies in WoS and Scopus databases, respectively, using the predetermined keywords. Later, automatic filters were used on the databases to exclude articles if they were not written in English and if they were book chapters, dissertations, or conference materials, leading to the exclusion of 247 papers. The two researchers then reviewed the topics to confirm that studies that were located on the Scopus database were also located on the WoS database, and 104 duplicates articles were removed. The titles and abstracts of each article were reviewed to ensure the eligibility of each title before retrieving them for analysis. In addition, where there is difficulty in relating the title or abstract to teacher well-being, the researchers reviewed the full text to confirm the article's eligibility. At this stage, 131 records (118 for title/abstract, 13 for full text) were excluded, leaving 172 studies for retrieval. In the process of retrieving the papers, 18 articles were not retrieved, resulting in the retrieval of 154 articles. At this stage, the researchers read the full text of each article, especially focusing on the introduction and methodology, and collectively agreed to remove some articles that were retrieved but could not meet the requirements for the review (non-empirical, marginal focus on teacher well-being, review study design, and involving pre-service teachers). At the end of this process, 58 papers were excluded, resulting in a total of 96 records being included from the Scopus and WoS databases.

Other search strategies were utilized to counteract the potential risk of publication bias and include high-quality articles that may not be located on the WoS and Scopus databases. First, a search on Google Scholar using the same keywords was conducted. Second, a citation search was also adopted to search for the articles mentioned in other reviews (Katsarou et al., 2023; Wilson et al., 2023; Zhang et al., 2024) and already-accessed articles (Collie, 2024; Cotson and Kim, 2024). As a result, seven papers were included, totaling 103 papers for the analysis, as shown in Figure 1. Table 1 presents some examples of papers included or excluded based.

**Table 1:** Inclusion and exclusion criteria.

**Criterion**	**Description**	**Example**
**Inclusion**		
Empirical	The results from these studies were based on empirical analysis (observation, interview measurements, and experiment)	Wang L. et al., 2022; Tsuyuguchi, 2023; Walter et al., 2023
Topic	The topic captures teacher wellbeing	Manasia et al., 2020; Forster et al., 2022; Gast et al., 2022
Major focus	The main focus of the study is teacher wellbeing	Granziera et al., 2022; Lau et al., 2022; Yang et al., 2022
In-service teachers	The participants included in the study are in-service teachers	Han et al., 2020; Kwon et al., 2022b; Liu et al., 2023
**Exclusion**		
Non-empirical	The results from these studies were not based on empirical studies	Hindman and Chor, 2023
Topic/abstract/full text	The topics, abstract, or full-text work did not focus on teacher wellbeing	Walker et al., 2004; Zhou and Ma, 2022
TWB marginal	In this type of study, teacher wellbeing was not the focus	Richter et al., 2022; Stark et al., 2022
Review	These studies were either systematic reviews or meta-analyses	Harris et al., 2020; Fan, 2021; Hascher and Waber, 2021; Yu et al., 2022
Pre-service teachers	Studies that investigated pre-service teachers as participants	Lee et al., 2022; Wang X. et al., 2022

### 2.3 Data extraction and analysis

The review adopted a quantitative descriptive technique to report the research trajectory on teacher well-being in the selected period. According to Zhang et al. (2024), the quantitative method effectively illustrates the shifts in study on topics over a specific period by showing the trajectory and patterns of research on issues throughout a particular period. To ensure a rigorous evaluation of methodological quality, each researcher independently coded 5% of studies randomly using predetermined key data points (Chinh et al., 2019). The coding was done utilizing a data analysis technique developed by the authors in Excel to analyze the publications that met the inclusion criteria. To ensure data reliability, the two researchers sorted each study according to the level of agreement (authors, publication date, and number of participants) before making a final decision (Whiting et al., 2003). In most cases, our discussion is focused on studies with lower agreement, and this process is presented in Supplementary Table 1.

## 3 Results

The results from this systematic review were obtained from 103 empirical studies on teacher well-being from the year 2020 to mid-February 2024. To ensure the authenticity of the included studies, all included articles were published in peer-reviewed journals. The articles published during this period were retrieved and analyzed according to the publication year, methodologies employed by the researchers, and the participants included in studies on the topic. Each article that is included for analysis is an original empirical study. Because of the restriction on articles to meet the objective of the study based on the inclusion criteria, many articles were excluded from the data.

### 3.1 Publications by year

**Figure 2:**
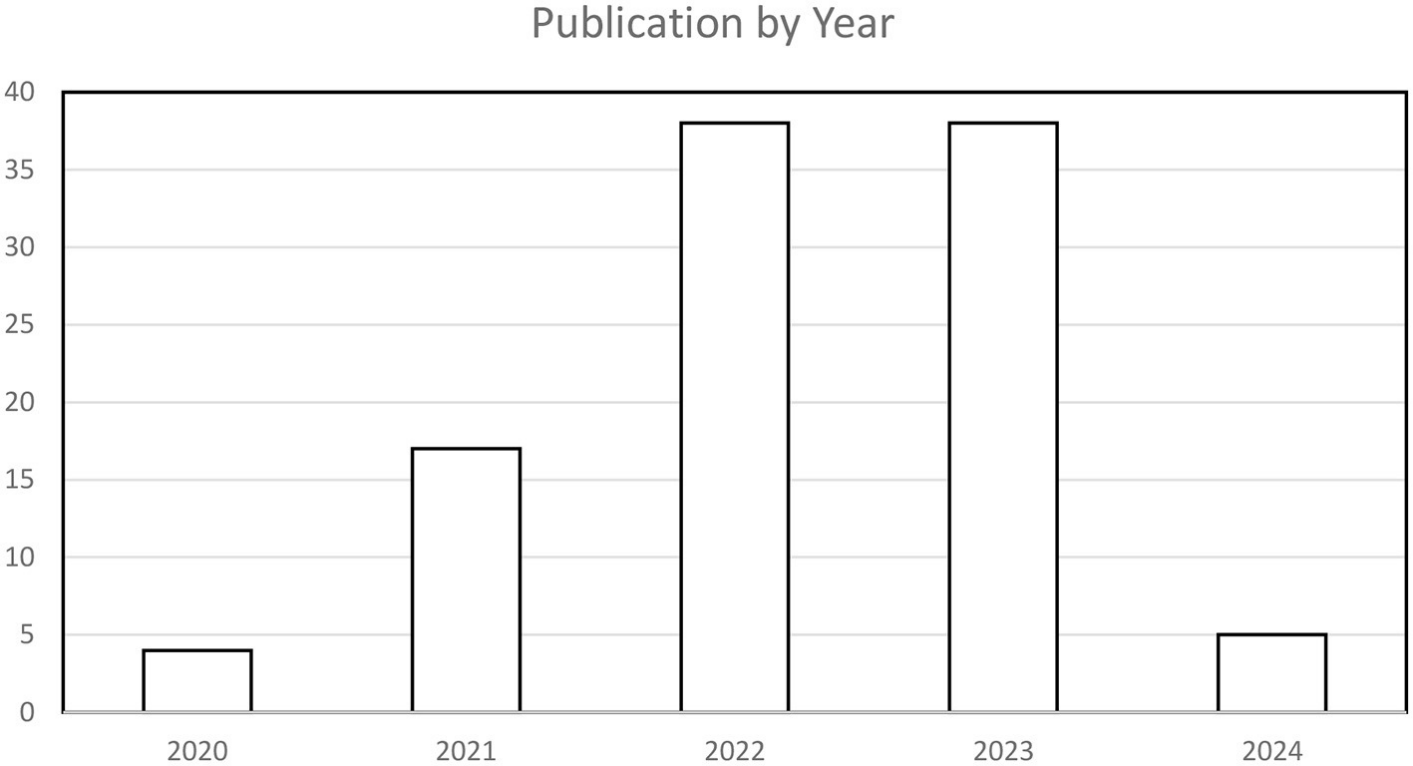
Publication trend from 2020 to 15th February 2024.

Regarding the number of publications per year from 2020 to mid-February 2024, a significant trend has been observed, indicating a notable expansion of interest in teacher well-being after 2020. Of the 103 published articles, only four (3.9%) were published in 2020 (e.g., Collie et al., 2020; Fatima and Wolf, 2020; Han et al., 2020; Manasia et al., 2020). There was a rapid expansion in 2021, with a total of 17 publications, representing 16.5% of the 103 included publications (e.g., Anderson et al., 2021; Herman et al., 2021; Montero-Marin et al., 2021; Ortan et al., 2021; Zhu et al., 2021). Articles published in 2022 form the largest segment of the corpus as the publications for that year increased to 39, representing 37.9% of the total publications (e.g., Forster et al., 2022; Kwon et al., 2022a; Shie and Chang, 2022; Thien and Lee, 2022; Yan et al., 2022; Yang et al., 2022). The number of publications for 2023 is very close to the record set in 2022, with 37 publications representing 35.9% of the total number of publications. Some of the studies published in 2023 are Fiegener and Adams (2023), Heikkila et al. (2023), Ostermeier et al. (2023), Shao (2023), Stone et al. (2023), and Walter et al. (2023). Although the dates for 2024 included only publications from January up to 15th February, it is notable that research has already started expanding on the topic, with six empirical studies representing 5.8% of the total publications (Cotson and Kim, 2024; Mendoza and Dizon, 2024; Vesely et al., 2024; Wang et al., 2024). Figure 2 presents a graphical representation of the yearly distribution of studies from 2020 to mid-February 2024.

### 3.2 Research methodology

**Figure 3:**
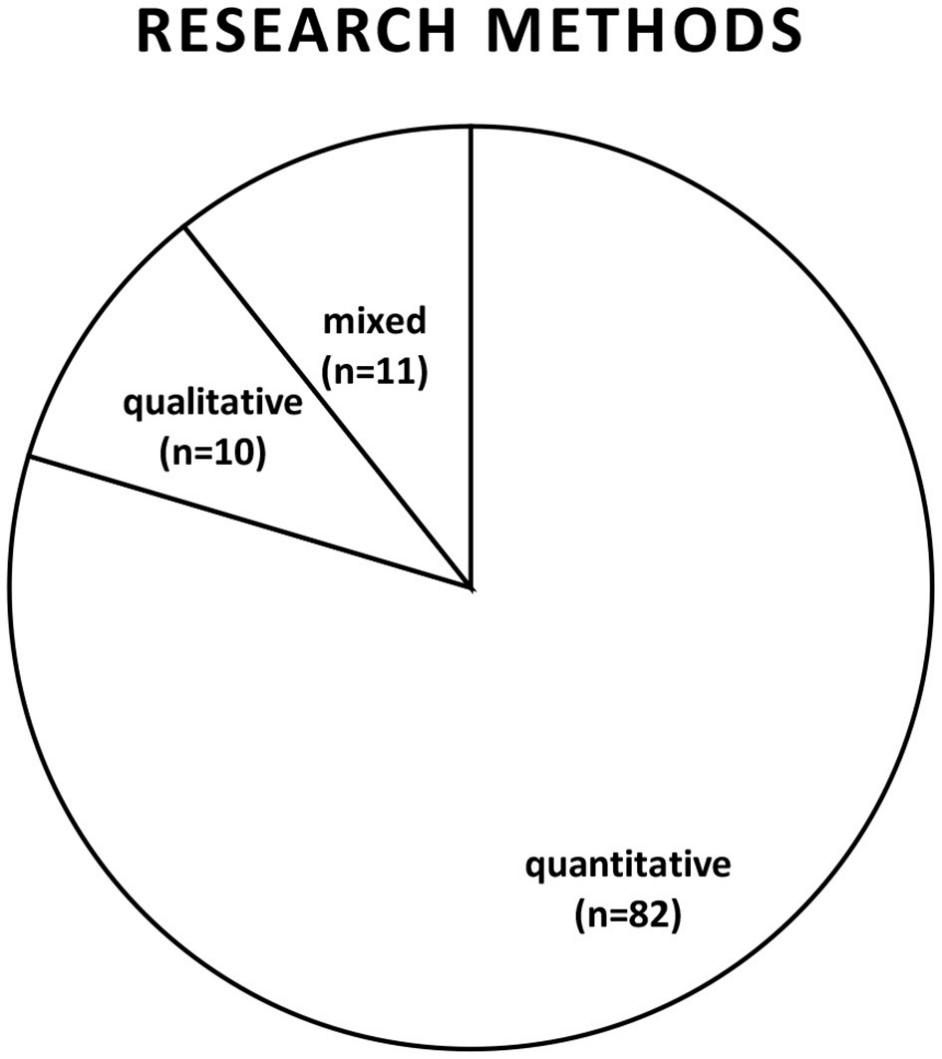
The distribution of research methodology from 2020 to 15th February 2024.

All articles were classified into three categories based on the research methods employed by researchers (qualitative, quantitative, and mixed methods). The researchers carefully sorted the articles according to the research methods adopted for each article. From 2020 to the middle of February 2024, out of the 103 published empirical studies retrieved, 10 of them (9.7%) adopted qualitative research methods in the form of interviews, case studies, document analysis, or a combination of them. Some studies adopted a qualitative research approach (Eloff and Dittrich, 2021; Haldimann et al., 2023). The quantitative method has been observed as scholars' expansively adopted this research methodology for TWB (for example, Liu et al., 2022; MacIntyre et al., 2022). Of the 103 published articles, 82 (79.6%) adopted the quantitative method, utilizing publicly available data or primary data collected through online surveys. Over the period, the mixed method appears to have started gaining acceptance by many scholars as they investigated teacher well-being with 11 (10.7%) articles (for example, Walter et al., 2023; Vesely et al., 2024). The adoption of qualitative methods increased in 2021, with most qualitative articles published in 2023. Figure 3 presents a graphical representation of the research method adopted over the period to investigate TWB.

### 3.3 Participants investigated

At this stage, the researchers carefully analyzed the participants' aspects of the Methods section of each article to identify the group of teachers targeted by TWB researchers over the period. A predetermined classification was used for this analysis in that the participants were grouped into preschool (early childhood) teachers, elementary (primary) school teachers, high school teachers (junior and senior high), university (all post-high schools) teachers, special education teachers, and teachers in general. Articles on general teachers in this regard refer to articles that were not specific to the group of teachers investigated in their research and articles that investigated teachers across various levels of education.

**Figure 4:**
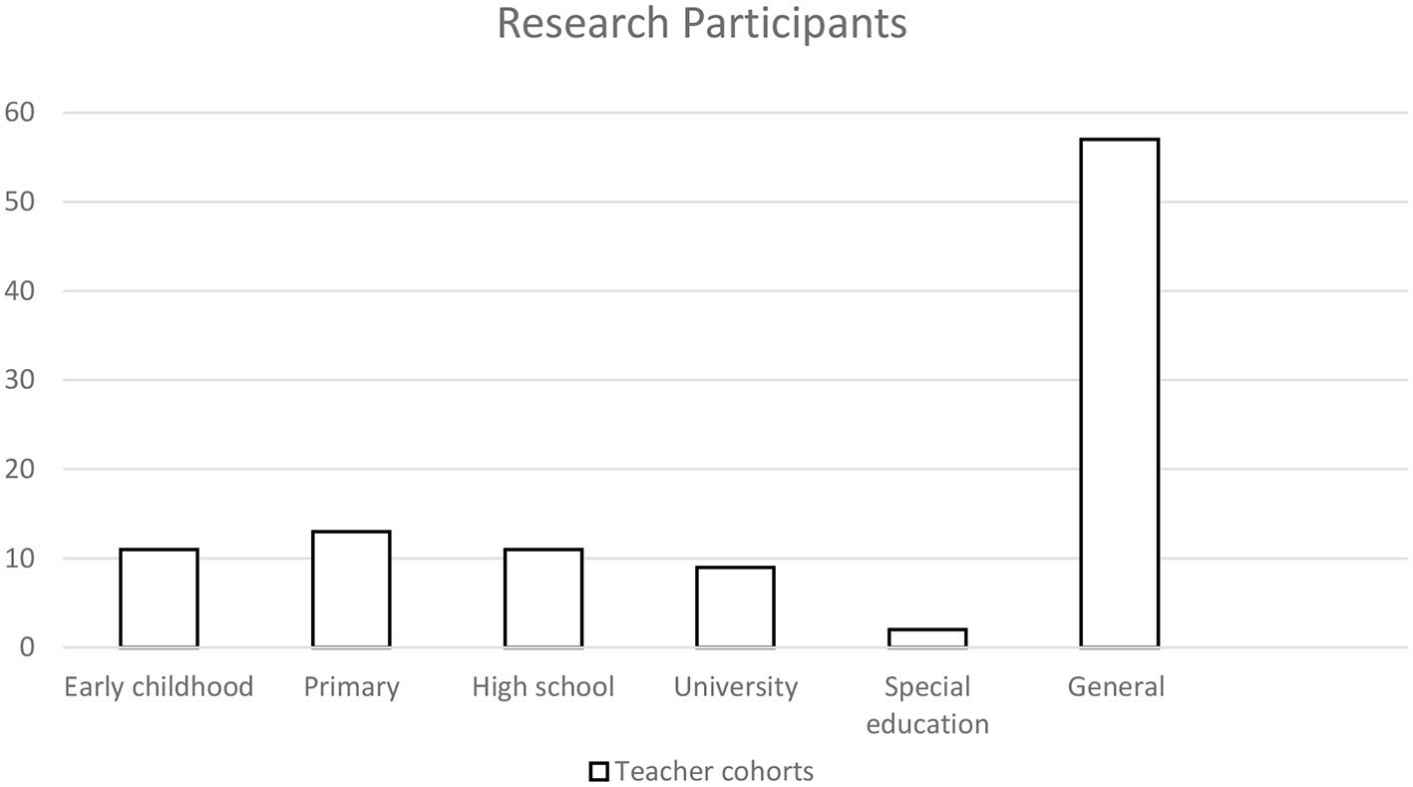
Distribution of participants investigated over the period.

After carefully matching the participants with each group, the reviewers identified that the majority of articles published within the stipulated period investigated teachers in general without focusing on a particular group. Of the articles included in this systematic review, 57 articles, representing 55.3%, investigated teachers in general. Many scholars have specifically investigated early childhood teachers' well-being in different parts of the world. Of the articles included in this systematic review, 11, representing 10.7% of the total included articles, focused on early childhood TWB. In addition, 13 articles, representing 12.6% of the total included articles, targeted primary school teachers for their investigation. Articles that targeted high school teachers were 11 in total, representing 10.7% of the 103 included articles. Publications on university teachers also started gaining importance, especially in 2022. Nine articles, representing 8.7% of the included articles, focused on university faculty members. Because of the peculiarity of special education, some scholars have also investigated teacher well-being, targeting only special education teachers. From 2020 to the beginning of mid-February 2024, a total of two papers focused on special education teachers, representing 1.9% of the total publications included in this review. Figure 4 shows a graphical representation of the distribution of articles among teaching cohorts.

## 4 Discussion

### 4.1 Summary of results

The systematic review is based on 103 empirical studies published from 2020 to mid-February 2024. The review aims to analyze the research distribution on teacher well-being during the selected period. Leveraging the quantitative review method, the researchers report an increase in studies on teacher well-being, especially in 2022 and 2023, reflecting the improved concern for TWB due to the COVID-19 pandemic. Though scholars have mostly utilized quantitative methods to investigate TWB (79.6%), attention has recently been given to qualitative and mixed methods. In addition, the study identified that many researchers investigated teachers in general (55.3%) rather than a specific group of teachers.

### 4.2 Publications by year

Concerning the number of publications per year from 2020 to mid-February 2024, it has been identified that research on teacher well-being has significantly increased. Publications on TWB peaked in 2022 and 2023. This trend can be attributed to the COVID-19 pandemic, as many countries reopened schools after 2020, leading to heightened interest in the well-being of teachers (Clayback and Williford, 2022; Flores et al., 2022; Blair et al., 2023; Collie, 2023), particularly concerning their mental health, retention, and attrition. This finding is consistent with a systematic review conducted by Zhang et al. (2024), which noted an acceleration in research on teacher well-being during and after the COVID-19 pandemic. The pandemic caused school closures, resulting in extended periods at home for teachers, and reopening schools often brought significant stress for both teachers and students. Addressing the emerging issues that may affect teachers has become critical (Lau et al., 2022). Given the increased importance of research on teacher well-being and the numerous studies investigating the factors affecting TWB, the trend of research should be adjusted to focus on investigating interventions or remedies to improve teacher well-being, reduce stress, and prevent teacher burnout.

### 4.3 Research methodology

On the research methodology employed by teacher well-being researchers, the quantitative research method appeared to be the most adopted methodology (79.6%) (Fernando Gallego-Nicholls et al., 2022; Ghasemi, 2022; Haines et al., 2022). Qualitative research and mixed methods started increasing in 2021 (Edwards et al., 2021; Falk et al., 2022). Advanced modeling approaches such as structural equation modeling, mediation, and moderation analysis were shown to be substantial in quantitative statistical procedures. Previous systematic reviews have also indicated that quantitative research methods were the most commonly used for studying TWB (Yu et al., 2022; Zhang et al., 2024). This prevalence can be attributed to the ability of quantitative methods to reach numerous respondents, particularly through publicly available data and surveys, enabling scholars to adopt these methods to investigate a large number of teachers. However, there is a growing need to conduct more qualitative or mixed-method research on the topic to gain a deeper understanding of TWB and intervention needs. Scholars may enhance their understanding of the factors influencing well-being by directly communicating with individuals. In the future research researchers should qualitatively gather information from various education authorities and teachers before determining the factors influencing TWB and identifying appropriate intervention measures to improve teacher well-being.

### 4.4 Participants investigated

The analysis indicates that there is limited research on special education teachers compared to studies focusing on general, early childhood, primary, high school, and university teachers. This gap is significant because Jeon et al. (2022) found that many teachers in special education either leave the field altogether or plan to do so due to the high rates of stress, burnout, and trauma they experience on the job. More research is needed to better understand the elements inherent in their profession that impact their well-being.

When participants were categorized according to their education level, primary school teachers appeared to have more publications than early childhood, high school, and university teachers, though the difference was insignificant. One possible explanation could be that primary education forms the foundation of higher education, and primary school teachers play a crucial role in students' education. Though not included in the research aims, the researchers found that research on English as a second language teachers is generally more specific in terms of subject specificity compared to teachers of other disciplines, such as Science, Technology, Engineering, and Mathematics (STEM) education (Han, 2022; Derakhshan et al., 2023; Shao, 2023). Moreover, none of the 103 explicitly included articles addressed STEM teachers. Because STEM teachers play an important role in preparing students for a technologically driven environment, research on their well-being is imperative. Stress from keeping up with the ever-changing standards of the curriculum and the particular demands of teaching STEM subjects can affect their mental health (Navarro-Espinosa et al., 2021). In addition to improving the quality of STEM education, understanding and addressing the factors that influence the well-being of STEM teachers can enhance their job satisfaction and retention rates. There is also a need to intensify TWB studies at both the university and early childhood education levels to ensure a balanced understanding of the topic.

### 4.5 Practical implication

The results of our systematic review have revealed several practical implications for researchers, policymakers in the field of education, school leaders, and organizations that support teachers. For instance, the increase in the number of publications, particularly in 2022, indicates a growing concern for teacher well-being, driven by the pandemic and its associated stressors. These stressors include the sudden shift to online teaching, challenges in maintaining work-life balance, and the need to adapt to new health and safety protocols.

Our findings highlight the significant influence of teacher well-being on education quality. Therefore, allocating resources to enhance the well-being of teachers, schools, and other educational institutions can foster a more favorable and efficient learning environment. This can be accomplished by implementing comprehensive support systems, including mental health services, professional development programs that prioritize resilience, and workload management measures to reduce stress and improve their overall well-being.

Furthermore, although there is a noticeable focus on incorporating qualitative and mixed approaches, the prevalence of quantitative methods, particularly surveys, indicates a substantial dependence on survey-derived data for evaluating well-being measures and results. Therefore, to maintain the attention of researchers in the field, it is necessary for governments and school leaders to conduct well-being surveys regularly to monitor the psychological and physical well-being of teachers. These surveys should be readily accessible to researchers conducting investigations. Researchers can use the survey data to create qualitative and mixed-method studies that provide insights into the specific stresses experienced by teachers, the coping strategies they use, and the most effective forms of support. This approach not only raises awareness of the intricate and diverse nature of teacher well-being but also facilitates a deeper understanding of the experiences, perspectives, and environmental factors that influence teachers.

Finally, researchers should prioritize including teachers in their studies, with a particular focus on addressing the unique needs of teachers in specific fields such as special education and STEM education. Exploring the distinct needs of teachers in particular fields can guide educational leaders in tailoring interventions and well-being initiatives to address these specific needs. This approach can enhance the efficacy of support programs, improve overall teacher job satisfaction, and increase teacher retention. For example, special education teachers may benefit more from interventions that incorporate stress management and coping skills tailored specifically to managing their unique classroom challenges, while university faculty might need support in balancing research and teaching tasks.

## 5 Conclusion

Research interest in teacher well-being has significantly increased from 2020 to mid-February 2024, as indicated by our systematic review of 103 articles. Since the onset of the COVID-19 pandemic, educational researchers have placed substantial emphasis on TWB. In 2022 and 2023, the impact of the pandemic on educational systems globally led to an increase in publications. Many researchers focused on investigating the relationship between teachers' physical and mental health during and after the pandemic. It was also observed that a shift toward evaluating therapies for TWB and their effects on job satisfaction and turnover started increasing in 2023. While the pandemic may have influenced this transformation, it also indicates that educational policy and practice are beginning to recognize the significance of TWB. Most of the studies conducted on teacher well-being in the selected years utilized quantitative research methodologies. However, there has been a notable increase in qualitative and mixed-methods research recently, indicating scholars' growing interest in a comprehensive understanding of TWB. While qualitative studies explore teachers' practical experiences and contextual influences on their well-being, mixed approaches provide statistical rigor and broader coverage. The complicated nature of teacher well-being requires a future approach that balances quantitative and qualitative methodologies. Studies focusing on general education teachers were more frequent than those focusing on special education teachers. To improve education systems and enhance TWB, a more detailed strategy is required to consider the specific needs of various teacher groups and utilize quantitative, qualitative, and mixed methods.

### 5.1 Limitations

The current review has one major limitation: it included studies published only from 2020 to mid-February 2024. This timeframe makes it difficult to understand how teacher well-being was explored prior to the COVID-19 outbreak. However, the review contributes new knowledge regarding the trends in TWB research, guiding future researchers in this field. Research on teacher well-being provides stakeholders with critical information about factors that may contribute to teacher attrition and offers suggestions for supporting teachers. Therefore, in the future research, TWB should be thoroughly examined by gathering input from various education authorities before identifying the factors that influence it. Moreover, future reviews should include publications from before, during, and after the COVID-19 pandemic to better understand the changes in the research trajectory over time.

## Data availability statement

The original contributions presented in the study are included in the article/Supplementary material, further inquiries can be directed to the corresponding author.

## Author contributions

MA: Conceptualization, Data curation, Formal analysis, Investigation, Methodology, Writing – original draft, Writing – review & editing. BZ: Conceptualization, Data curation, Methodology, Writing – original draft, Writing – review & editing.

## Supplementary material

Supplementary Table 1 Study characteristics.
