# Supplementary figures and images for: Systematic review of teacher well-being research during the COVID-19 pandemic
Source: Front Psychol. 2024 Sep 6;15:1427979. doi: 10.3389/fpsyg.2024.1427979 (PMC11414468; doi:10.3389/fpsyg.2024.1427979)

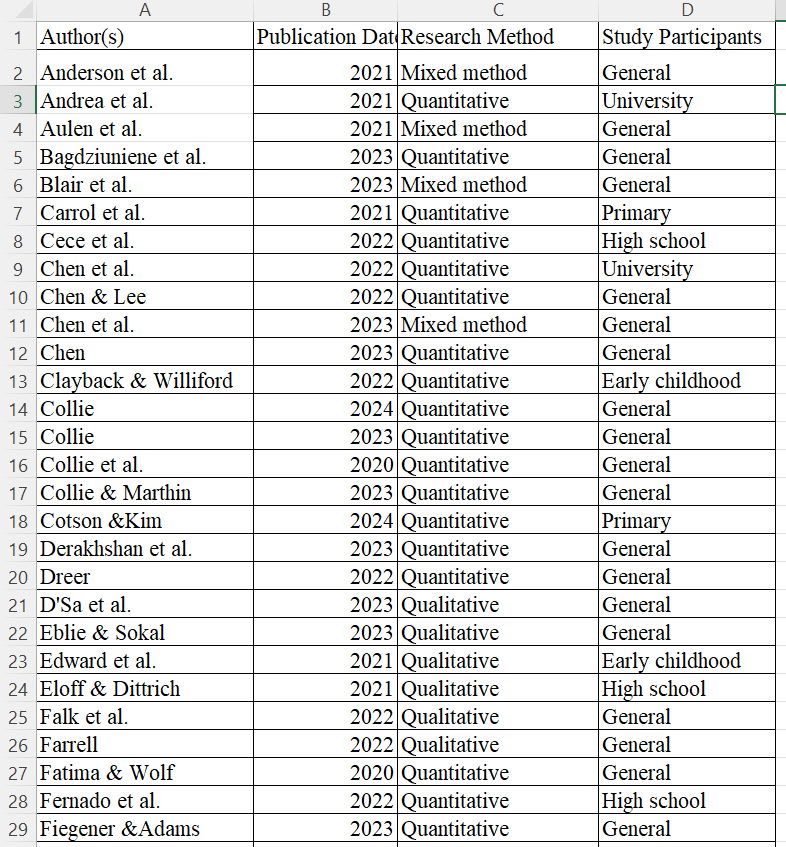


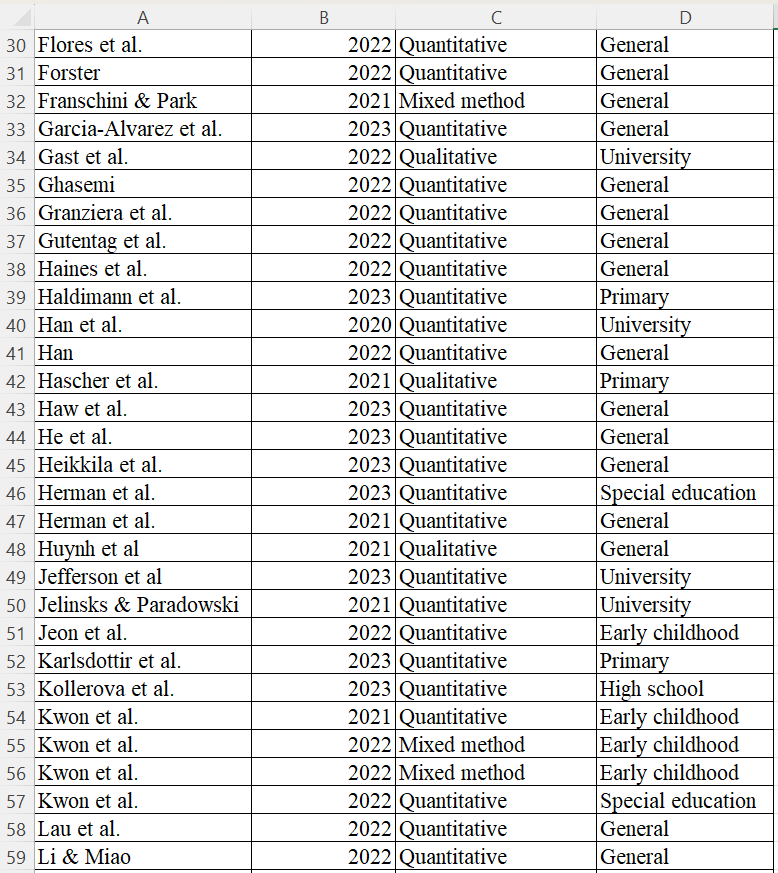


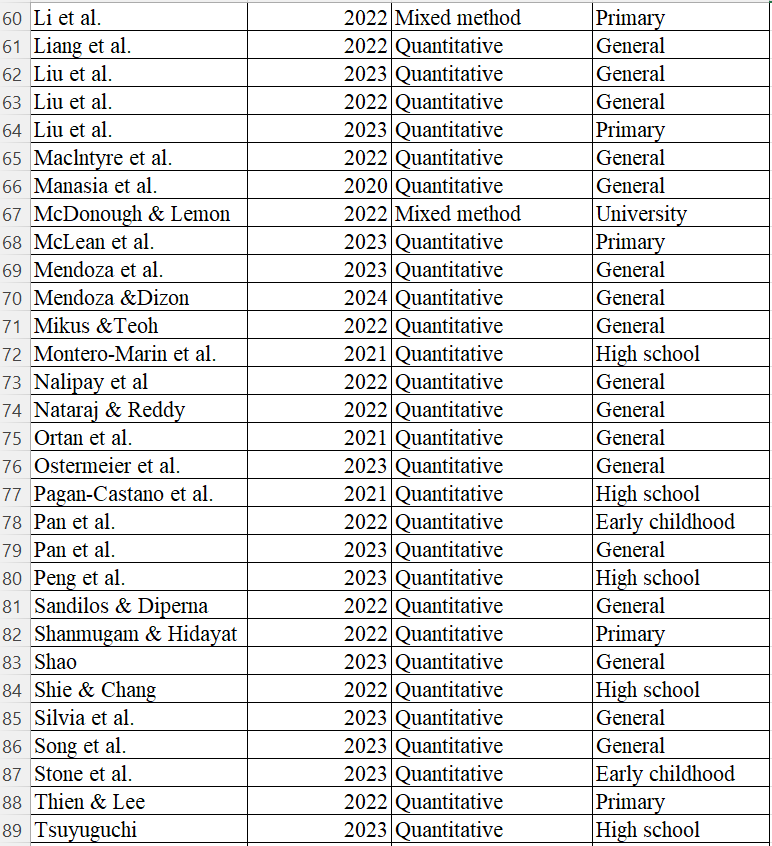


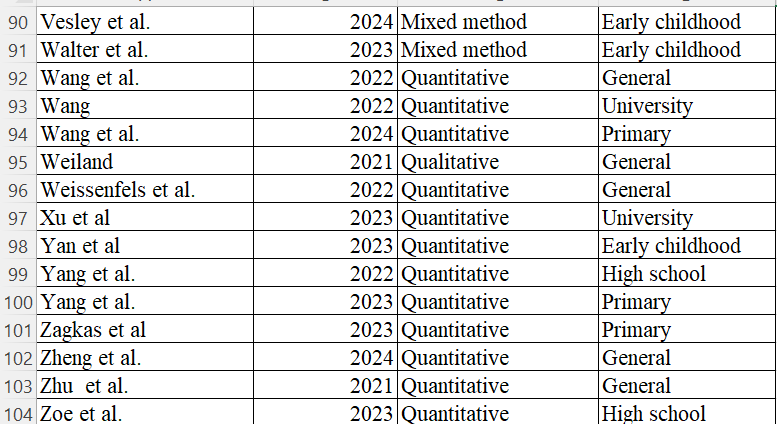

Supplement: Supplementary Table 1 — Study characteristics. [file Table_1.DOCX]
